# Effects of Rice Bran Oil on the Intestinal Microbiota and Metabolism of Isoflavones in Adult Mice

**DOI:** 10.3390/ijms130810336

**Published:** 2012-08-17

**Authors:** Motoi Tamura, Sachiko Hori, Chigusa Hoshi, Hiroyuki Nakagawa

**Affiliations:** National Food Research Institute, the National Agriculture and Food Research Organization, Tsukuba, Ibaraki 305-8642, Japan; E-Mails: vets@affrc.go.jp (S.H.); chig3@affrc.go.jp (C.H.); hironkgw@affrc.go.jp (H.N.)

**Keywords:** rice bran oil, equol, daidzein, mice, intestinal microbiota

## Abstract

This study examined the effects of rice bran oil (RBO) on mouse intestinal microbiota and urinary isoflavonoids. Dietary RBO affects intestinal cholesterol absorption. Intestinal microbiota seem to play an important role in isoflavone metabolism. We hypothesized that dietary RBO changes the metabolism of isoflavonoids and intestinal microbiota in mice. Male mice were randomly divided into two groups: those fed a 0.05% daidzein with 10% RBO diet (RO group) and those fed a 0.05% daidzein with 10% lard control diet (LO group) for 30 days. Urinary amounts of daidzein and dihydrodaidzein were significantly lower in the RO group than in the LO group. The ratio of equol/daidzein was significantly higher in the RO group (*p* < 0.01) than in the LO group. The amount of fecal bile acids was significantly greater in the RO group than in the LO group. The composition of cecal microbiota differed between the RO and LO groups. The occupation ratios of *Lactobacillales* were significantly higher in the RO group (*p* < 0.05). Significant positive correlation (*r* = 0.591) was observed between the occupation ratios of *Lactobacillales* and fecal bile acid content of two dietary groups. This study suggests that dietary rice bran oil has the potential to affect the metabolism of daidzein by altering the metabolic activity of intestinal microbiota.

## 1. Introduction

Much attention has recently focused on the health benefits of soy-based foods. These health benefits have been largely attributed to isoflavones. Daidzin, genistin, daidzein (the aglycone of daidzin), and genistein (the aglycone of genistin) are the most common isoflavones found in soy products. Isoflavones are a class of phytoestrogens. Phytoestrogens are defined as compounds that exert estrogenic effects on the central nervous system, induce estrus, and stimulate growth of the genital tract in female animals [1]. Human gastrointestinal bacteria seem to play an important role in isoflavone metabolism [2,3]. Equol is a metabolite of daidzein and is produced by intestinal flora [4]. Equol is more effective than daidzein in competing with 3*H*-estradiol to bind to the estrogen receptor, suggesting that equol has a higher affinity for this receptor [5]. It has also been suggested that the ability to produce equol or equol itself is closely related to the lower incidence of prostate cancer [6].

Equol is also being studied in terms of its role in reducing the incidence and frequency of menopausal symptoms [7]. Equol inhibited bone loss and fat accumulation in estrogen-deficient osteoporotic mice [8]. Thus, equol is an important bacterial metabolite in the gut. However, inter-individual variations in equol production have been identified. Only 30% to 50% of humans are equol producers [9]. It has been reported that the frequency of equol producers among vegetarians is 59%, similar to the reported frequency in Japanese adults consuming soy and much higher than that among non-vegetarian adults (25%), suggesting that dietary components other than soy influence *S*-equol synthesis by intestinal bacteria [10]. Rowland *et al.* [11] demonstrated that humans who excrete high concentrations of equol consume less fat and more carbohydrates as a percentage of energy than humans who excrete low concentrations of equol. The bioavailability of isoflavonoids seems to be affected by the composition of the diet. The beneficial effects of rice bran oil (RBO) have recently been reported. The hypolipidemic effect of RBO is not entirely explained by its fatty acid composition. RBO has a greater content of unsaponifiables [12], which also lower cholesterol compared with most vegetable oils [13]. RBO seems to affect the hyperinsulinaemic response. It has also been reported that RBO may improve lipid abnormalities, reduce the atherogenic index, and suppress the hyperinsulinaemic response in rats with streptozotocin/nicotinamide-induced type 2 diabetes mellitus [14]. It has been reported that the RBO group had a higher high-density-lipoprotein cholesterol concentration and greater excretion of fecal neutral sterols and bile acid than did the control group [14]. On the other hand, bile acid inhibition of intestinal anaerobic organisms has been reported [15]. Intestinal anaerobe inhibition by bile acids may represent a regulatory mechanism of the intestinal microbiota [15,16]. RBO seems to affect gut function and intestinal microbiota. Intestinal microbiota seem to play an important role in equol production [4]. We tested the hypothesis that dietary rice bran oil changes the metabolism of isoflavonoids and intestinal microbiota in mice. The aim of the present study was to investigate these effects in mice.

## 2. Results and Discussion

### 2.1. General Observations

No significant differences were observed between the RO and LO groups in final body weight (g) (RO, 38.0 ± 0.6; LO, 35.4 ± 1.1), food consumption (g/day) (RO, 4.5 ± 0.1; LO, 4.5 ± 0.1), visceral fat (g) (RO, 1.92 ± 0.17; LO, 1.69 ± 0.25), cecal contents (RO, 0.18 ± 0.03; LO, 0.19 ± 0.02), amount of feces (g/day) (RO, 0.36 ± 0.02; LO, 0.36 ± 0.02) or liver weight (g) (RO, 1.62 ± 0.02; LO, 1.53 ± 0.07).

### 2.2. Urinary Isoflavonoids

**Figure 1:**
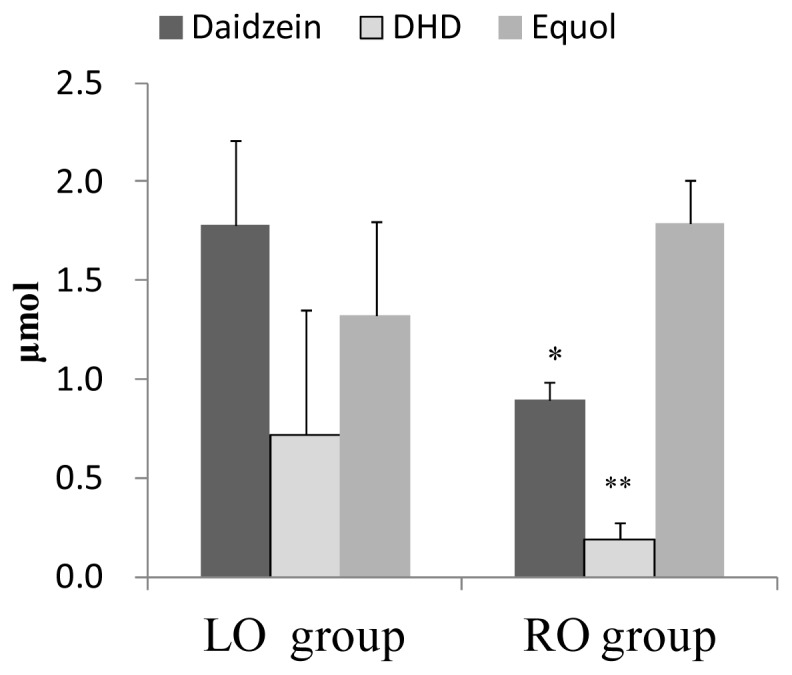
Amounts of urinary isoflavonoids (aglycones + metabolites) of mice in the LO group and the RO group. Values are means ± SE (*n* = 7). * Significantly different (*p* < 0.05) from the LO group. ** Significantly different (*p* < 0.01) from the LO group. The data were analyzed using *t*-test analysis (equol) or Mann-Whitney rank sum test (daidzein, DHD (dihydrodaidzein)). Statistical significance was reached with a *p* value of less than 0.05.

The diet containing rice bran oil significantly affected the metabolism of daidzein compared with the control diet. The urinary amounts of daidzein were significantly higher in the LO group than in the RO group (*p* < 0.05) (Figure 1). The urinary amounts of dihydrodaidzein (DHD) were significantly higher in the LO group than in the RO group (*p* < 0.01) (Figure 1). Average urinary amounts of equol tended to be higher in the RO group than in the LO group. However, no significant differences were observed between the urinary amounts of equol in the RO and LO groups. The ratio of equol/daidzein was significantly higher in the RO group (2.07 ± 0.28) (*p* < 0.01) than in the LO group (0.74 ± 0.24). Dihydrodaidzein (DHD), a bacterial metabolite of the widespread isoflavone daidzein [2], is proposed as a precursor of equol [2]. It has been demonstrated that daidzein is converted to dihydrodaidzein (DHD), benzopyran-4,7-diol, 3-(4-hydroxyphenyl) and equol by human fecal bacteria [17]. Dihydrodaidzein (DHD) has been postulated as an intermediate in the formation of equol. Metabolites of daidzein may be important because of the different biological effects of these compounds. Thus, intestinal microbiota appear to play an important role in the biological activities of isoflavones. In our results, urinary amounts of daidzein and DHD was significantly greater in LO group than in RO group. However, urinary equol was tended to be high in the RO group. Metabolic activity of intestinal microbiota against isoflavonoids might be changed by rice bran oil. Tocotrienol and oryzanol are major unsaponifiable compounds (UC) in RBO. Oryzanol has ferulic acid ester. On the other hand, it has been reported that ferulic acid was metabolized by intestinal microbiota [18]. *Lactobacillus johnsonii* that displayed strong ferulic acid esterase activity in stool samples from diabetes-resistant biobreeding rats [19]. *Lactobacillus fermentum* NCFB 1751 showed the highest level of ferulic acid esterase activity [20]. Thus, intestinal microbiota including lactobacilli affect the metabolism of ferulic acid ester. Oryzanol in RBO might affect the metabolism of intestinal microbiota resulting in the change of the metabolic activity of daidzein.

### 2.3. Plasma Total Cholesterol, Triglyceride, Phospholipids, and Plasma Glucose

At the end of the diet feeding period, the mice were anesthetized with diethylether and blood samples were taken from the abdominal aorta and placed in heparinized tubes. The plasma was separated from whole blood by centrifugation and used for analysis of plasma triglyceride, total cholesterol, phospholipids, and glucose. No significant differences in the plasma cholesterol (RO, 195.7 ± 24.3 mg/dL; LO, 184.7 ± 19.3 mg/dL), or plasma phospholipids concentrations (RO, 291.8 ± 21.6 mg/dL; LO, 268.4 ± 17.3 mg/dL) were observed between the two groups. No significant differences between the two groups were observed for plasma glucose (RO, 220.7 ± 19.7 mg/dL; LO, 225.6 ± 33.1 mg/dL). However, plasma triglyceride concentrations tended to be lower in the LO (LO, 139.4 ± 10.2 mg/dL) than in the RO (RO, 179.0 ± 22.0 mg/dL) group (*p* = 0.129). It has been reported that RBO showed a significant (15%–17%) reduction in cholesterol absorption and a significant (30%) increase in neutral sterol excretion [21]. However, in our experiment, no significant differences in plasma cholesterol were observed between the RO and LO groups. Both diets contained daidzein. It was reported that an isoflavone aglycone-rich extract without soy protein attenuated atherosclerosis development in cholesterol-fed rabbits [22]. Dietary daidzein contained in the experimental diet affected the plasma lipids in both the RO and LO groups.

### 2.4. Effects of Diet on Cecal Flora and Fecal Amounts of Bile Acid of Mice

**Figure 2:**
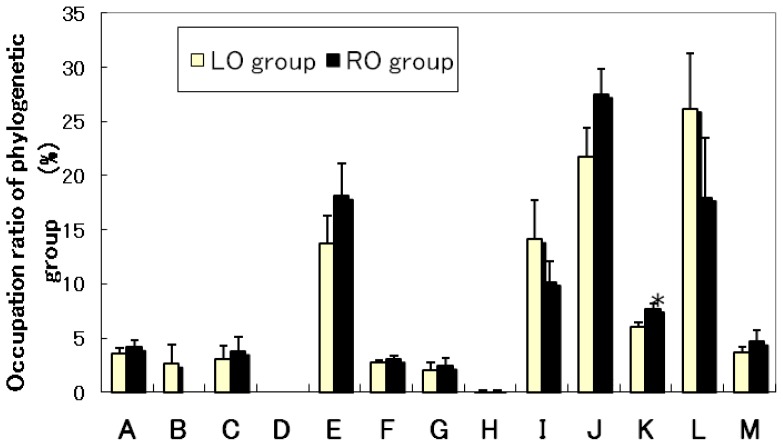
Composition of cecal intestinal microbiota of mice in the RO and LO groups. OTUs (operational taxonomic units), which correspond to either T-RFs (terminal restriction fragments) or T-RF clusters, detected by T-RFLP analysis. Values are means ± SE (*n* = 7). * Significantly different (*p* < 0.05) from the LO group. The data were analyzed using *t*-test analysis. The letters correspond to the following phylogenetic bacterial groups: (**A**) *Bacteroides*, *Clostridium* cluster IV (OTUs 370); (**B**) *Clostridium* cluster IV (OTUs 168, 749); (**C**) *Clostridium* cluster IX, *Megamonas* (OTUs 110); (**D**) *Clostridium* cluster XI (OTUs 338); (E) *Clostridium* subcluster XIVa (OTUs 106, 494, 505, 517, 754, 955, 990); (**F**) *Clostridium* cluster XI, *Clostridium* subcluster XIVa (OTUs 919); (**G**) *Clostridium* subcluster XIVa, *Enterobacteriales* (OTUs 940), H: *Clostridium* cluster XVIII (OTUs 423, 650); (**I**) *Bacteroides* (OTUs 469, 853); (**J**) *Bifidobacterium* (OTUs 124); (**K**) *Lactobacillales* (OTUs 332, 520, 657); (**L**) *Prevotella* (OTUs 137, 317); (**M**) Others.

**Figure 3:**
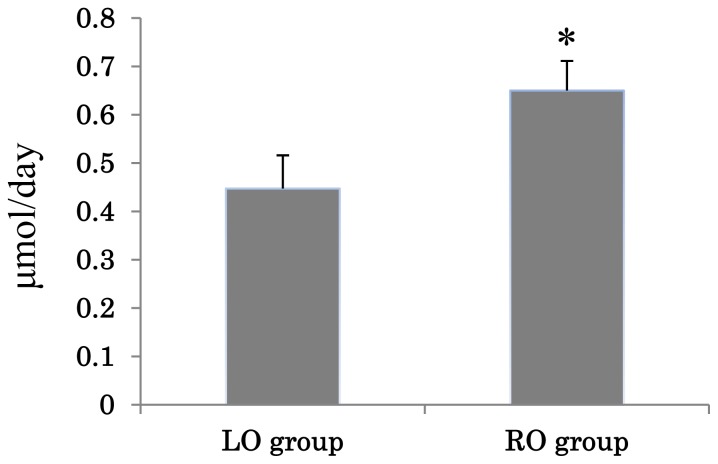
The amount of fecal bile acids (μmol/day) from the mice in the LO group and the RO group. Values are means ± SE (*n* = 7). The data were analyzed using *t*-test analysis. * Significantly different from the LO group (*p* < 0.05).

It has been confirmed that human intestinal microbiota predominantly consist of members of approximately ten phylogenetic bacterial groups and that these bacterial groups can be distinguished by the T-RFLP system developed by Nagashima *et al.* [23,24]. Figure 2 depicts the compositions of the cecal flora, which differed between the two dietary groups. The occupation ratios of *Lactobacillales* were significantly higher in the RO group (*p* < 0.05). The RO diet significantly affected the amounts of fecal bile acids. The amount of fecal bile acids was significantly greater in the RO group than in the LO group (*p* < 0.05) (Figure 3). Pearson product-moment correlation coefficient between the occupation ratios of *Lactobacillales* and fecal bile acid content of two dietary groups were analyzed. Significant positive correlation (*r* = 0.591) was observed between the occupation ratios of *Lactobacillales* and fecal bile acid content of two dietary groups. Diet composition affects the gut microbial composition to a larger extent than previously thought [25], and intestinal microbiota strongly affect isoflavone metabolism *in vivo* [26]. Diet composition may affect equol production from daidzein in the gut by modifying the metabolic activity and/or composition of intestinal microbiota. In our results, there were significant differences in the composition of microbiota between the RO and LO groups. The occupation ratios of *Lactobacillales* were significantly higher in the RO group. In our experiment, fecal amounts of bile acids were significantly higher in the RO group than in the LO group. It has been reported that bile acids affect the intestinal bacteria [27]. Pearson product-moment correlation coefficient between the occupation ratios of *Lactobacillales* and fecal bile acid content of two dietary groups were analyzed. Significant positive correlation (*r* = 0.591) was observed between the occupation ratios of *Lactobacillales* and fecal bile acid content of two dietary groups. Bile-salt hydrolase is the enzyme responsible for deconjugation of bile acid resulting in free bile salts. Bile salt hydrolase has been detected in several lactic acid bacteria species [28,29]. Out of 297 strains of *Lactobacillus* spp. screened for bile-salt hydrolase activity on plates, 191 were positive [29]. It has been reported that the human isolate *Lactobacillus fermentum* KC5b was able to maintain viability for 2 h at pH 2 and to grow in a medium with 4000 mg of bile acids per liter [30]. Thus, *Lactobacillales* play an important role in bile acid metabolism in the gut. Increased bile acid excretion in the RO group may have affected the composition of microbiota, resulting in the higher occupation ratios of *Lactobacillales*. On the other hand, Oryzanol are major unsaponifiable compounds (UC) in RBO. It has been reported that ferulic acid was metabolized by intestinal microbiota [18]. Oryzanol has ferulic acid ester. It has been shown that microencapsulated *Lactobacillus fermentum* 11976 cells can efficiently break down a ferulic acid-containing substrate [31]. It has been found that among the intestinal bacteria tested, *Lactobacillus acidophilus* exhibited the highest level of activity with respect to feruloylated arabinose ester [32]. Oryzanol might affect the metabolism of *Lactobacillus* resulting in the increased occupation ratios of *Lactobacillales* in the RO group. In general *Lactobacillales* produce short chain fatty acids. Increased occupation ratio of *Lactobacillales* may have increased the lactate production resulting in increasing the butyrate by butyrate-producing bacterium. Lactate could be a major precursor for butyrate synthesis by the intestinal microbiota [33,34]. Short chain fatty acids seem to affect the metabolism of daidzein. It has been reported that butyric acid increases the conversion ratio of daidzein to equol in equol-producing bacteria [35]. Some lactic acid bacteria affect the equol production in the fecal microbiota. *Lactobacillus fermentum* ATCC9338 altered the equol production status in the *in vitro* incubation of daidzein with fecal microbiota of mice [36]. It has been reported that *L. rhamnosus* JCM2771 increased the equol production in the *in vitro* incubation of daidzein with fecal microbiota from a male equol producer [37]. It has been demonstrated that daidzein is converted to dihydrodaidzein (DHD) [17]. DHD has been postulated as an intermediate in the formation of equol. Urinary amounts of daidzein and DHD was significantly greater in LO group than in RO group. However, urinary equol was tended to be high in the RO group. Changes in productivity of SCFA in the gut might be caused by *Lactobacillales*, and these changes might have affected the increased equol production and decreased DHD production in the fecal flora of the RO group, resulting in the different metabolism between the two dietary groups.

Recently, much attention has been focused on the relation between intestinal flora and obesity. Studies on human volunteers have revealed that obesity is associated with changes in the relative abundance of the two dominant bacterial divisions, the Bacteroidetes and the Firmicutes [38]. A significantly higher *Lactobacillus* species concentration in obese patients than in lean controls (*p* = 0.0197) or anorexic patients (*p* = 0.0332) has been reported [39]. Obesity in rats fed the high fat diet was accompanied by higher *Lactobacillus*/*Enterococcus* and lower numbers of *Bacteroides*/*Prevotella* and with permanently higher alkaline phosphatase activity [40]. Pearson product-moment correlation coefficient between the occupation ratios of *Lactobacillales* and final body weight of two dietary groups were analyzed. In our experiment, positive correlation (*r* = 0.41) was observed between the occupation ratios of *Lactobacillales* and final body weight of two dietary groups. The occupation ratios of *Lactobacillales* might be related with body weight.

We could not identify the species of bacteria that increased by the increased fecal amounts of bile acids as the limited resolution of T-RFLP. However, this is the first report on the effects of dietary RBO on the metabolism of daidzein in mice.

## 3. Experimental Section

### 3.1. Materials

Daidzein and equol were purchased from LC Laboratories (Woburn, MA, USA). β-Glucuronidase type H-5 was obtained from Sigma (St. Louis, MO, USA).

### 3.2. Treatment of Animals

Male Crj:CD-1 mice (6 weeks old) were purchased from Charles River Japan, Inc. (Kanagawa, Japan). All mice were specific pathogen-free and were housed in conventional conditions in our laboratory. The mice were randomly divided into two groups of seven animals each. The animals were housed individually in suspended stainless-steel cages with wire mesh bottoms in a room kept at 24 ± 0.5 °C and a relative humidity of 65%, with 12-h periods of light and dark. They were fed an AIN-93M diet for 7 day. After 7 day, the diet was replaced with a 0.05% daidzein with 10% RBO diet (RO diet) or a 0.05% daidzein with 10% lard diet (LO diet), and the mice received the RO or LO diet for 30 days. Twenty-three days after beginning to feed the experimental diet, all animals were moved to metabolic cages (Tecniplast S.p.a., Buguggiate (Va), Italy). The animals were housed individually in these metabolic cages. Urine was collected for 47 h from all mice. Urinary amounts of isoflavonoids were measured. The purified diet and water were provided ad libitum. Table 1 presents the composition of each diet. The RO diet (control diet) contained 0.05% daidzein and 10% RBO; the LO diet contained 0.05% daidzein and 10% lard.

Body weight and food consumption were measured and feces were collected during the experiment. Feces were dried with a freeze dryer (FD-1000; Tokyo Rikakikai Co., Ltd., Tokyo, Japan) for 24 h. The trap cooling temperature was −45 °C. Weights of freeze-dried feces were measured during the experiment. After the experimental diet-feeding period, the mice were anesthetized with diethylether, and blood samples were taken from the abdominal aorta and placed in heparinized tubes. All mice were euthanized with diethylether. The plasma was separated from whole blood by centrifugation and stored at −80 °C for later analysis of plasma triglyceride, total cholesterol, phospholipids, and glucose. The liver, visceral fat, and cecal contents were collected. Cecal contents were stored at −80 °C until HPLC analysis for later terminal restriction fragment length polymorphism (T-RFLP) analysis of intestinal microbiota. The liver samples and visceral fat were weighed. All procedures involving mice in this study were approved by the Animal Care Committee of the National Food Research Institute in Japan and were performed in accordance with the “Guidelines for Animal Care and Experimentation” of the National Food Research Institute of the National Agriculture and Food Research Organization in Japan.

### 3.3. Measurement of Plasma Cholesterol, Triglyceride, Phospholipids, and Plasma Glucose

The following tests were performed with Wako kits obtained from Wako Pure Chemical Industries Ltd., Osaka, Japan. Total plasma cholesterol concentrations were measured using a cholesterol E-test Wako kit based on cholesterol oxidase [41]. Plasma triglyceride concentrations were measured using a triglyceride E-test Wako kit based on the glycerol-3-phosphate oxidase method [42]. Plasma phospholipid concentrations were measured using a phospholipid C-test Wako kit based on the choline oxidase method [43]. The plasma glucose concentrations were measured using a glucose C2-test Wako kit based on the mutarotase glucose oxidase method.

### 3.4. Measurements of Fecal Weight and Fecal Amounts of Bile Acid

All feces were collected during the experiment. Weights of feces were measured. Feces were then dried with a freeze dryer (FD-1000; Tokyo Rikakikai Co., Ltd., Tokyo, Japan) for 24 h. The trap cooling temperature was −45 °C. After drying, weights of freeze-dried feces were measured. Feces were milled with a food mill (TML17; TESCOM Co., Ltd., Tokyo, Japan) for 30 s. Fecal amounts of bile acid were measured by the method of Kanamoto *et al.* [44]. A total of 50 mg of feces were suspended in a glass test tube with 2.5 mL of 99.5% ethanol, vortexing for 30 s, incubation for 1 h at 65 °C, and centrifugation at 3000 rpm for 10 min at 4 °C. The supernatants were transferred to a glass test tube. The same volume as that used in the first extraction of 99.5% ethanol was added to the sediment, and the procedure was repeated. The supernatants from both extractions were pooled in the same glass test tube and dried at 65 °C gassing with N_2_ gas. After drying, 0.5 mL of 90% ethanol was added to the residue and vortexed for 30 s. Total bile acid concentrations were measured with a total bile acid test (Wako Pure Chemicals, Osaka, Japan) according to the manufacturer’s instructions.

### 3.5. Analysis of Urinary Isoflavonoids

A total of 200 μL of urine was added to 200 μL of β-glucuronidase H-5 solution (35 mg/mL; Sigma, MO, USA) in 0.2 M sodium acetate buffer (pH 5.0). Next, the mixture was incubated at 37 °C in a water bath for 3 h, followed by treatment with 400 μL of ethyl acetate, vortexing for 30 s, and centrifugation at 5000*g* for 10 min at 4 °C. The supernatants were transferred to an eggplant-type flask. The same volume as that used in the first extraction of ethyl acetate was added to the sediment, and the procedure was repeated. The supernatants from both extractions were pooled in the eggplant-type flask and evaporated completely using a rotary evaporator. The sample was then dissolved in 400 μL of 80% methanol and filtrated through a 0.2-μm filter. Filtrates were used for HPLC analysis. For HPLC analysis, we injected 20 μL of each preparation into a 250 × 4.6-mm Capcell Pak MG C18 5-μm column (Shiseido, Tokyo, Japan). To detect isoflavonoids, a photodiode array detector (MD-1515; JASCO, Co., Ltd., Tokyo, Japan) was used to monitor the spectral data from 200 to 400 nm for each peak. To measure the isoflavonoids, we used daidzein and equol as standard samples. We used the spectral data at 254 nm to quantify daidzein and the spectral data at 280 nm to quantify equol and DHD. The mobile phase consisted of methanol/acetic acid/water (35:5:60, *v*/*v*/*v*). The running conditions of HPLC were a column temperature of 40 °C and a flow rate of 1 mL/min.

### 3.6. DNA Extraction from Cecal Contents

DNA extractions from cecal contents were conducted according to Matsuki’s method [45]. Cecal samples (20 mg) were washed three times by suspending them in 1.0 mL of phosphate-buffered saline and centrifuging each preparation at 14,000*g* to remove possible PCR inhibitors. Following the third centrifugation, the cecal pellets were resuspended in a solution containing 0.2 mL of phosphate-buffered saline and 250 μL of extraction buffer (200 mM Tris-HCl, 80 mM EDTA; pH 9.0) and 50 μL of 10% sodium dodecyl sulfate. A total of 300 mg of glass beads (diameter, 0.1 mm) and 500 μL of buffer-saturated phenol were added to the suspension, and the mixture was vortexed vigorously for 180 s using a Micro Smash MS-100 (TOMY SEIKO Co., Ltd., Tokyo, Japan) at 4700 rpm. Following centrifugation at 14,000 *g* for 5 min, 400 μL of the supernatant was collected. Phenol-chloroform-isoamyl alcohol extractions were then performed, and 250 μL of the supernatant was subjected to isopropanol precipitation. Finally, the DNA was suspended in 1 mL of Tris-EDTA buffer. The DNA preparation was adjusted to a final concentration of 10 μg/mL in TE and checked by 1.5% agarose gel electrophoresis.

### 3.7. PCR Conditions and Restriction Enzyme Digestion

The PCR mixture (25 μL) was composed of Ex Taq buffer, 2 mM of Mg^2+^, and each deoxynucleoside triphosphate at a concentration of 200 μM. The amount of cecal DNA was 10 ng. The primers used were 5′ FAM-labelled 516f (5′-TGCCAGCAGCCGCGGTA-3′) and 1510r (5′-GGTTACCTTGTTACGACTT-3′) at a concentration of 0.10 μM, template DNA, and 0.625 U of DNA polymerase (TaKaRa EX Taq; Takara Bio Inc., Otsu, Japan). This process was carried out using a PCR system (Dice system; Takara Bio Inc.). Amplification was performed with one cycle at 95 °C for 15 min, followed by thirty cycles at 95 °C for 30 s, 50 °C for 30 s, 72 °C for 1 min, and finally one cycle at 72 °C for 10 min. The amplification products were subjected to gel electrophoresis in 1.5% agarose followed by ethidium bromide staining. The PCR products were purified using spin columns (QIAquick; Qiagen KK, Tokyo, Japan) according to the manufacturer’s instructions. The purified DNA was treated with 2 U of *Bsl*I (New England Biolabs) for 3 h at 55 °C [24].

### 3.8. T-RFLP Analysis

T-RFLP analysis is based on PCR amplification of a target gene. The amplification is performed with one primer whose 5′ end is labelled with a fluorescent molecule. The mixture of amplicons is then subjected to a restriction reaction using a restriction enzyme. Following the restriction reaction, the mixture of fragments is separated using either capillary or polyacrylamide electrophoresis in a DNA sequencer, and the sizes of the different terminal fragments are determined by the fluorescence detector. We used this T-RFLP analysis in our experiment. The fluorescently labelled terminal restriction fragments (T-RFs) were analysed by electrophoresis on an automated sequence analyser (ABI PRISM 310 Genetic Analyzer; Applied Biosystems) in GeneScan mode. The restriction enzyme digestion mixture (2 μL) was mixed with 0.5 μL of size standards (MapMarker 1000; BioVentures, Inc.) and 12 μL of deionized formamide. The mixture was denatured at 96 °C for 2 min and immediately chilled on ice. The injection time was 30 s for analysis of T-RFs from digestion with *Bsl*I. The run time was 40 min. The lengths and peak areas of T-RFs were determined with GeneMapper software. The predominant operational taxonomic units (OTUs), which correspond to either T-RFs or T-RF clusters, were detected in the T-RFLP profiles and used to identify phylogenetic groups of intestinal microbiota [24,25].

### 3.9. Statistics

Data are expressed as the mean ± standard error (SE). All data were analysed using Sigma Plot 11 (Systat Software, Inc., CA, USA). The data were analysed with the *t*-test or Mann-Whitney rank sum test. Statistical significance was reached with a *p*-value < 0.05.

## 4. Conclusions

In conclusion, urinary amounts of daidzein and dihydrodaidzein were significantly lower in the RO group than in the LO group. The amount of fecal bile acids was significantly greater in the RO group than in the LO group. The occupation ratios of *Lactobacillales* were significantly higher in the RO group (*p* < 0.05). Significant positive correlation (*r* = 0.591) was observed between the occupation ratios of *Lactobacillales* and fecal bile acid content of two dietary groups. This study suggests that dietary rice bran oil has the potential to affect the metabolism of daidzein by altering the metabolic activity of intestinal microbiota.

## Figures and Tables

**Table 1:** Composition of the experimental diet.

Ingredient (g/kg diet)	AIN-93M	LO diet	RO diet
Cornstarch	465.692	405.186	405.186
Casein	140	140	140
α-Cornstarch	155	155	155
Sucrose	100	100	100
Soybean oil	40	-	-
Lard	-	100	-
Rice bran oil	-	-	100
Cellulose	50	50	50
Mineral mix	35	35	35
(AIN-93M-Mix)	-	-	-
Vitamin mix	10	10	10
(AIN-93-Mix)	-	-	-
l-Cystine	1.8	1.8	1.8
Choline bitartrate	2.5	2.5	2.5
Tert-butylhydroquinone	0.008	0.014	0.014
Daidzein a	-	0.5	0.5

a Purchased from LC Laboratories (Woburn, MA, USA).
